# Thymoquinone Inhibits Proliferation and Induces Apoptosis in Immortalized Keratinocytes via Upregulation of p53 Expression

**DOI:** 10.1155/drp/9764336

**Published:** 2025-12-04

**Authors:** Somaye Karimi, Narges Fallahi, Seyedeh Sindokht Hosseini, Mohammad Fereidouni, Fahime Ghasemi, Atena Mansouri, Nafiseh Erfanian, Mehdi Shakibaie, Mitra Rafiee

**Affiliations:** ^1^Student Research Committee, Birjand University of Medical Sciences, Birjand, Iran; ^2^Department of Immunology, School of Medicine, Cellular and Molecular Research Center, Birjand University of Medical Sciences, Birjand, Iran; ^3^Department of Medical Biotechnology, School of Medicine, Pharmaceutical Science Research Center, Birjand University of Medical Sciences, Birjand, Iran; ^4^Cellular and Molecular Research Center, Birjand University of Medical Sciences, Birjand, Iran; ^5^Department of Pharmaceutics and Nanotechnology, School of Pharmacy, Pharmaceutical Sciences Research Center, Birjand University of Medical Science, Birjand, Iran

**Keywords:** apoptosis, keratinocyte cells, P53, proliferation, thymoquinone

## Abstract

**Introduction:**

Psoriasis, as a common inflammatory skin disease, is characterized by hyperproliferation of epidermal keratinocytes and induction of an inflammatory response. Apoptosis induction and prevention of the proliferation of keratinocytes can help to treat and manage this disease. Thymoquinone (TQ), a bioactive compound with antioxidant and anti-inflammatory properties, has also been reported as a natural antitumor agent. This study aimed to evaluate the effects of TQ on proliferation and apoptosis in human keratinocyte cells (HaCaT).

**Methods:**

HaCaT cells were treated with increasing concentrations of TQ (1, 2, 4, 6, 8, 16, 32, and 64 μg/mL), and cell viability was assessed using the MTT assay. Apoptosis was analyzed via flow cytometry using Annexin V-FITC/PI staining. Expression levels of p53, Bax, and BCL-xl genes were measured by real-time PCR.

**Results:**

TQ significantly reduced cell viability in a dose-dependent manner, with an IC50 of 11.64 μg/mL after 72 h. Flow cytometry revealed a marked increase in early apoptotic cells following treatment with 8 μg/mL TQ (41.00% ± 5.04%) compared to control (17.8% ± 2.26%, *P* ≤ 0.001). Gene expression analysis showed significant upregulation of p53, while Bax and BCL-xl levels showed no significant changes.

**Conclusion:**

TQ induces apoptosis in human HaCaT cells primarily through p53-dependent pathways, suggesting its potential as a therapeutic agent for skin-related disorders.

## 1. Introduction

The skin, as a physical, chemical, and immunological barrier, carries out several vital tasks [1]. The physical and functional integrity of the skin is related to three layers: the epidermis as the top layer, the dermis as the middle layer, and the hypodermis as the deepest layer of the skin [2, 3]. Keratinocytes, as the main cells of the epidermis, are involved in maintaining the functional, structural, and physical integrity of the skin. However, constant evidence has shown that keratinocytes play complementary roles that go beyond the creation of a protective barrier against external factors. In fact, after stimulation, these cells actively participate in the initiation and adjustment of inflammatory skin reactions with the synthesis and release of cytokines, and respond to various inflammatory reactions [2].

Psoriasis belongs to the category of autoimmune and hypersensitive disorders and is a chronic inflammatory skin condition [4]. The hallmarks of this disease are the infiltration of several immune cell types and epidermal hyperplasia [5]. Psoriasis is initiated and maintained in large part by keratinocytes. These cells affect dendritic cell activation in the first phase. Th1 and Th17 cell activation occurs by triggered dendritic cell cytokines, and these cells will also activate keratinocytes by the production of different inflammatory cytokines [6, 7]. Additionally, several chemokines and cytokines produced by activated keratinocytes will contribute to the strengthening of inflammation during the subsequent stage of the disease [8]. Psoriasis is characterized by excessive proliferation and resistance of keratinocytes to apoptosis [9]. Gene expression profiling plays a critical role in elucidating the molecular mechanisms underlying apoptosis. Key regulators such as p53, Bax, and BCL-xL are frequently assessed to determine the balance between proapoptotic and antiapoptotic signals in response to various stimuli. Evaluating the transcriptional changes of these genes provides insight into the pathways activated during cell death and can help identify potential therapeutic targets [10]. Therefore, identifying bioactive compounds capable of modulating apoptosis-related genes may open new therapeutic avenues in psoriasis.

“Black Cumin” is indigenous to Eastern European and West Asian nations like Iran. It has been used in traditional medicine to treat many diseases, including high blood pressure, diabetes, asthma, bronchitis, and inflammation [11]. Thymoquinone (TQ), as a major ingredient of Black Cumin, has been shown to have anti-inflammatory and high antioxidant properties [12]. Anti-inflammatory properties of TQ have caused it to be useful for the treatment of inflammatory illnesses like colitis, rheumatoid arthritis, allergic asthma, and allergic encephalitis [13]. TQ prevents the synthesis of proinflammatory cytokines like IL-1 and IL-6 and obstructs the maturation of dendritic cells [14, 15]. It effectively inhibits the NF-κβ signaling pathway, which generates inflammatory cytokines [13].

Some psoriasis treatments rely on keratinocyte cell apoptosis induction and the prevention of proliferation. Vitamin D3 and glucocorticoids induce apoptosis and prevent keratinocyte development. Retinoids also function as substances that normalize keratinocyte proliferation, making them a therapeutic alternative [9]. Although TQ is known for its anti-inflammatory and proapoptotic properties, it remains unclear whether it can effectively regulate keratinocyte proliferation and apoptosis in psoriasis. Therefore, the present study aimed to investigate the effects of TQ on cell proliferation, apoptosis, and the expression of apoptosis-related genes (p53, Bax, and BCL-xL) in HaCaT cells.

## 2. Materials and Methods

### 2.1. Cell Culture and Treatment

The human HaCaT cells were cultured in RPMI-1640 medium (Gibco, Thermo Fisher Scientific, USA) supplemented with 10% heat-inactivated fetal bovine serum (FBS; Gibco, Thermo Fisher Scientific, USA) and 1% penicillin/streptomycin (Gibco, Thermo Fisher Scientific, USA). Cells were incubated at 37°C and 5% CO_2_ in a humidified incubator.

### 2.2. Evaluation of Cytotoxicity Effects of TQ on HaCaT Cells Using MTT Assay

Cytotoxic effects of TQ (Sigma, Cat. No. 274666), dissolved in DMSO (final concentration ≤ 0.1%, nontoxic to HaCaT cell), were evaluated using the MTT assay. HaCaT cells were seeded (1 × 10^4^ cells per well) in a 96-well plate and treated with different concentrations of TQ (1, 2, 4, 6, 8, 16, 32, and 64 μg/mL) for 72 h. The cells were then treated with 3-[4,5-dimethylthiazol-2-yl]-2,5-diphenyltetrazolium bromide (MTT; Sigma) to a final concentration of 5 mg/mL and incubated for 4 h at 37°C. Formazan crystals were dissolved in 150 μL DMSO, and absorbance was measured at 570 nm using a microplate reader (Epoch, BioTek Instruments, Winooski, VT). All experiments were performed in triplicate in three independent experiments.

### 2.3. Evaluation of Apoptosis Induced by TQ on HaCaT Cells

HaCaT cells (1 × 10^5^ cells/well) were seeded in six-well plates and incubated with 4 and 8 μg/mL of TQ for 72 h. After treatment, the cells were harvested using trypsin-EDTA, washed twice with cold PBS, and resuspended in binding buffer. Apoptosis was assessed using a propidium iodide/FITC Annexin V staining kit (Padza Pajoh, Iran), followed by analysis with a flow cytometer (CyFlow Cube 6). The percentage of apoptotic cells was then determined using FlowJo V7.

### 2.4. Gene Expression Analysis by Real-Time qPCR

HaCaT cells were treated with 8 μg/mL TQ for 48h prior to RNA extraction. To determine mRNA expression levels, total RNA was extracted from HaCaT cells using an RNA extraction kit (Total RNA Extraction Kit, Pars Toos, Iran) according to the manufacturer's instructions. One microgram of total RNA was reverse-transcribed to cDNA using a cDNA synthesis kit (cDNA Reverse Transcription Kit, Pars Toos, Iran). The mRNA expression levels of BCL-xl, BAX, and p53 were quantified by real-time qPCR using a SYBR Green kit (SYBR Green Ampliqon Kit, Ampliqon, Denmark) on an ABI Real-Time PCR System (Applied Biosystems, USA). Primer sequences are listed in Table 1. Thermal cycling conditions were 95°C for 10 min, followed by 40 cycles of 95°C for 10 s, 60°C for 30 s, and 72°C for 30 s. Reactions were performed in technical triplicates and normalized to GAPDH. Relative expression was calculated using the 2^−ΔΔCT method. Data are presented as mean ± SD, and a *p*-value < 0.05 was considered statistically significant.

### 2.5. Statistical Analysis

Data analysis was performed using SPSS software (Version 26) and Prism 9. The quantitative results are reported as mean ± standard deviation (SD). The results were compared with a one-way analysis of variance (ANOVA) and *t*-test. *P*-value < 0.05 was considered as a significant level.

## 3. Results

### 3.1. Cytotoxicity Effects of TQ on HaCaT Cells

**Figure 1:**
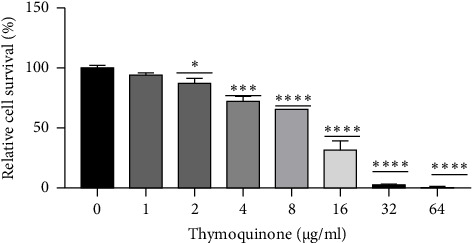
The outcomes of the MTT assay. The *x*-axis indicates various concentrations of thymoquinone, and the *y*-axis represents the percentage of cell viability. The concentrations tested ranged from 1 to 64 μg/mL. *P* values significance is defined as ^∗^*p* < 0.05, ^∗∗^*p* < 0.01, ^∗∗∗^*p* < 0.001, and ^∗∗∗∗^*p* < 0.0001.

The results of the current study showed that different concentrations of TQ significantly decreased cell viability in a dose-dependent manner compared to the control group. The IC50 value after 72 h was determined to be 11.64 (Figure 1). Based on these findings, concentrations of 4 and 8 μg/mL were selected for apoptosis analysis, while the 8 μg/mL concentration was further used for gene expression studies.

### 3.2. Effects of TQ on HaCaT Cells' Apoptosis

**Figure 2:**
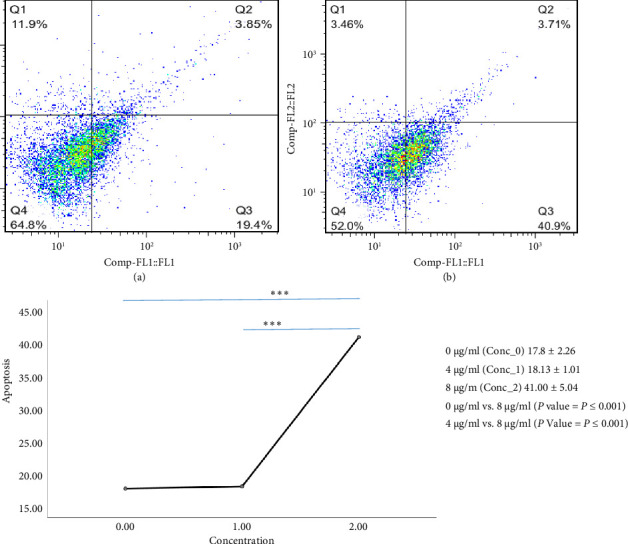
The population has been evaluated using two FITC dyes attached to Annexin and PI (FL1: Annexin+ and FL2: PI+). Annexin+ and PI-cell populations have been analyzed (early apoptotic cells). Plot A shows the control population that was not affected by TQ. Plot B shows the cell population after the effect of a TQ concentration of 8 μL/mL. Panel D shows the changes in the percentage of apoptotic cells in the presence of TQ compared to the control. The results are reported as mean ± SD. The significance level is considered to be ≤ 0.05.

Flow cytometry analysis demonstrated that TQ treatment significantly induced apoptosis in HaCaT cells in a dose-dependent manner. The percentage of early apoptotic cells was 17.8% ± 2.26% in untreated cells (0 μg/mL), 18.1% ± 1.0% at 4 μg/mL, and 41.0% ± 5.0% at 8 μg/mL. Statistical analysis showed significant differences between 0 and 8 μg/mL (*P* < 0.001) as well as between 4 and 8 μg/mL (*P* < 0.001) (Figure 2).

### 3.3. Effects of TQ on Apoptosis-Related Gene Expression in HaCaT Cells

**Figure 3:**
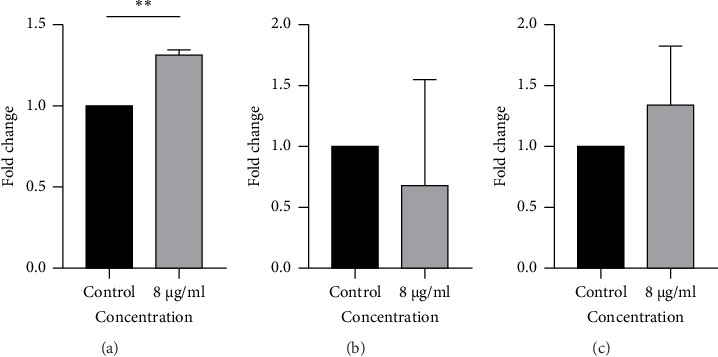
Fold change in relative expression of apoptosis-related genes after treatment with 8 μg/mL. (A) p53; (B) BCL-xL; (C) Bax. A significant upregulation was observed only for p53 (*p* < 0.01). *P* values significance is defined as ^∗^*p* < 0.05, ^∗∗^*p* < 0.01, ^∗∗∗^*p* < 0.001, and ^∗∗∗∗^*p* < 0.0001.

The effects of TQ treatment on apoptosis-related gene expression were evaluated by real-time PCR (Figure 3). In the case of p53 (Figure 3(a)), treatment with 8 μg/mL significantly increased the gene expression compared to the control group (mean ± SD: 1.321 ± 0.027 vs. 1.000 ± 0.000; *p* < 0.01). For BCL-xl (Figure 3(b)), a reduction in expression was observed in the treated group (0.6918 ± 0.8602) compared to the control (1.000 ± 0.000), although this difference did not reach statistical significance (*p* > 0.05). Regarding Bax (Figure 3(c)), treatment resulted in an elevation of expression (1.350 ± 0.4808 vs. 1.000 ± 0.000 in control), but the change was not statistically significant (*p* > 0.05). Collectively, these findings suggest that the treatment markedly induces p53 expression, while exhibiting a nonsignificant trend toward downregulation of BCL-xl and upregulation of Bax, potentially indicating a shift in the apoptotic balance.

## 4. Discussion

The current research demonstrated that TQ hinders growth and triggers cell death in human keratinocytes. Psoriasis, as a common inflammatory skin disease, is characterized by hyperproliferation of epidermal keratinocytes and induction of an inflammatory response. T cells, inflammatory cytokines such as IFN-γ, IL-17, and TNFα, directly and indirectly affect keratinocytes and promote abnormal proliferation and differentiation of keratinocytes [16]. HaCaT cells have been obtained for the first time from the unafflicted skin area near a melanoma, and they have been rendered immortal in the culture media following over 140 passages [17]. Because keratinocyte cells exhibit heightened proliferation and resistance to apoptosis in psoriasis, the HaCaT cell line is used as an appropriate cell line related to psoriasis disease research. Indeed, substances that inhibit proliferation and promote apoptosis in keratinocyte cells are of therapeutic significance and warrant consideration for treatment strategies. Recently, there has been significant research on compounds that inhibit the proliferation and induce apoptosis of keratinocytes, some of which have even made their way into the commercial market [9]. Aquaporin 3 (AQP3) is a protein found in tissues like the skin, respiratory tract, kidney, and certain cancers. Its levels are typically elevated in skin hyperplasia disorders, and the upregulation of AQP3 is associated with increased keratinocyte proliferation and epidermal hyperplasia. In 2014, Zhouwei and colleagues showed that natural polyphenols like resveratrol can reduce the proliferation of keratinocytes by decreasing AQP3 levels. This mechanism suggests that these compounds could be beneficial for inhibiting skin hyperplasia disorders [18]. In 2007, Dujic and collaborators illustrated that low doses of curcumin, in combination with visible light, can effectively inhibit proliferation and induce apoptosis in skin keratinocytes. This finding suggests that this approach could serve as an effective treatment for hyperproliferative skin diseases [19]. The current study introduces TQ as a novel compound with the potential to inhibit the proliferation of keratinocyte cells. Our results showed that TQ induced apoptosis in the HaCaT cell line.

Multiple studies have confirmed TQ's ability to impede proliferation and prompt apoptosis across diverse cell lines and diseases through different mechanisms. In 2021, Raut and collaborators confirmed that TQ stimulated apoptosis in SK-MEL-28 human melanoma cells. Their study revealed that this compound diminished the JAK/STAT3 signaling pathway and downregulated associated genes such as cyclin D1, D2, D3, and Survivin [19]. Numerous studies have suggested that TQ induces apoptosis by multiple mechanisms, including upregulation of the proapoptotic protein p53, downregulation of the antiapoptotic protein BCL-xL, and elevation of BAX expression, thereby promoting apoptosis through the mitochondrial pathway. The present findings revealed that TQ induced apoptosis in HaCaT cells primarily through p53 activation, without significant modulation of Bax or BCL-xL. This observation suggests a p53-dependent response that appears less reliant on the canonical mitochondrial Bax/BCL-xL imbalance in these cells; however, direct assessment of mitochondrial events (e.g., cytochrome-c release, mitochondrial membrane potential, caspase-9 activation) was not performed and would be required to conclusively demonstrate a mitochondria-independent pathway [20].

Interestingly, although many anticancer agents induce apoptosis via mitochondrial disruption and changes in Bax/BCL family ratios, TQ has been reported to modulate BCL family members variably across cell systems—decreasing antiapoptotic proteins (BCL-2/BCL-xL) in some contexts while acting through alternative (e.g., ROS- or death-receptor-linked) mechanisms in others—indicating a dose- and cell-type-dependent mode of action [21].

This cell-type specificity is particularly relevant for keratinocytes, in which BCL-xL is a central prosurvival BCL-2 homolog and is strongly regulated by epidermal signaling; therefore, reporting BCL-xL (rather than BCL-2) more accurately reflects antiapoptotic control in epidermal cells [22].

Finally, the selective upregulation of p53 at lower, noncytotoxic concentrations of TQ may favor regulated keratinocyte turnover without extensive tissue damage, a property that is desirable in hyperproliferative skin disorders. Restoration or activation of p53 signaling has been shown to promote epidermal differentiation and suppress aberrant proliferation in keratinocyte models, supporting the therapeutic relevance of the present observation [10].

Furthermore, this substance notably enhances the generation of reactive oxygen species (ROS), HSP70, and caspases 3 and 8, pivotal in the apoptotic pathway [20, 21]. While the present study demonstrates that TQ effectively inhibits proliferation and induces apoptosis in HaCaT cells, further investigations are needed to fully elucidate its mechanisms of action. In particular, additional analyses could explore the potential involvement of mitochondrial pathways, ROS production, and caspase activation. Moreover, extending these studies to in vivo models and other keratinocyte types would provide a more comprehensive understanding of TQ's therapeutic potential in hyperproliferative skin disorders. Collectively, these findings support TQ as a promising candidate for the development of novel treatments for hyperproliferative skin disorders and provide a foundation for further mechanistic and translational studies.

## 5. Conclusion

The present study indicates that TQ effectively inhibits proliferation and induces apoptosis in HaCaT cells, highlighting its potential as a therapeutic agent for managing hyperproliferative skin disorders such as psoriasis.

## Figures and Tables

**Table 1:** Primer sequences used for quantitative real-time PCR analysis of apoptosis-related genes.

Gene symbol	Forward primer	Reverse primer
P53	CAGCACATGACGGAGGTTGT	TCATCCAAATACTCCACACGC
BCL-xL	GAGACTCAGTGAGTGAGCAGGTG	GCTTGTAGGAGAGAAAGTCAACC
Bax	TGCCAGCAAACTGGTGCTC	AACCACCCTGGTCTTGGAT
GAPDH (reference)	AAGCTCATTTCCTGGTATG	CTTCCTCTTGTGCTCTTG

## Data Availability

The datasets used and/or analyzed during the current study are available from the corresponding author on reasonable request.
